# Dopamine D2R Agonist-Induced Cardiovascular Effects in Healthy Male Subjects: Potential Implications in Clinical Settings

**DOI:** 10.1155/2014/956353

**Published:** 2014-01-22

**Authors:** Khalid Abou Farha, Corine Baljé-Volkers, Wim Tamminga, Izaak den Daas, Sandra van Os

**Affiliations:** ^1^QPS Netherlands B.V., Petrus Campersingel 123, 9713 AG Groningen, P.O. Box 137, 9700 AC Groningen, The Netherlands; ^2^Synthon B.V., Microweg 22, 6545 CM Nijmegen, The Netherlands

## Abstract

Dopamine D2 receptor agonists represent a first line treatment option in young patients with signs and symptoms of idiopathic Parkinson's disease. An association between the use of D2 receptor agonists in Parkinson's disease patients and heart failure has been reported. The identification of the underlying mechanism is needed to minimize the resultant cardiovascular morbidity. In a phase I clinical trial, a D2 receptor agonist (pramipexole) was administered to 52 healthy male subjects following a dose escalation scheme. Serial measurements of resting blood pressure, heart rate, and derived parameters including pulse pressure, pulsatile stress, and rate pressure product were analysed. Statistically significant and clinically relevant increases in most of the assessed parameters were found. Ten subjects were removed prematurely from the trial because of clinically significant increases in blood pressure and/or heart rate requiring immediate intervention with IV rescue medications including a selective **β**-1 blocker. The observed drug-related changes in vital signs were of clinical relevance and might explain some of the cardiovascular morbidity reported in patients receiving D2 receptor agonist in clinical settings. We suggest that the additional use of a **β**-1 blocking agent might mitigate the risk of cardiovascular morbidity among patients receiving long-term D2 receptor agonists.

## 1. Introduction

Dopamine (D) is a naturally occurring catecholamine neurotransmitter that mediates its biologic functions by 2 main classes of G protein-coupled receptors: D1-class which includes D1 and D5 receptor (R) subtypes and D2-class which includes D2, D3, and D4 receptor subtypes [1, 2].

It has been reported that nigral and striatal D2Rs have a key function in controlling locomotor behaviour and motor skills [1–3] and that ablation of striatal D2Rs leads to Parkinsonian-like locomotor behaviour [4–6]. This substantiates the use of D2R agonists in the treatment of Parkinson's disease (PD). In this context, D2R agonists are first line treatment option in de novo and young PD patients with mild-to-moderate symptoms. D2R agonists are also used in combination with levodopa, the gold standard treatment for PD, to delay the development of disabling motor complications in advanced stages of the disease [6–8]. In addition, D2R agonists are not metabolised by oxidative pathways and therefore do not lead to cytotoxic free radical formation that cooccurs with levodopa administration [7]. Unfortunately, the use of D2R agonists might be associated with cardiovascular (CV) complications including orthostatic hypotension (OH) and heart failure (HF). A recent European multicentre study found a relationship between the use of D2-like R agonist (pramipexole) in patients with PD and HF especially in early phase of therapy [9]. The identification of precipitating factors for this serious CV morbidity is a key to developing appropriate strategies aiming to prevent or minimize potential D2R agonist-induced CV complications.

In this report we describe our CV findings in a phase I clinical trial in 52 healthy male volunteers that evaluated a nonergot selective D2-like R agonist (pramipexole) administered orally in an escalating dose level design, over a dose range of 0.125–4.5 mg once daily.

## 2. Material and Methods

A cohort of 52 healthy nonsmoking male subjects aged between 18 and 55 years (mean 32 years) were recruited to participate in a phase I clinical trial purposed to evaluate the safety, tolerability, and pharmacokinetics of a high-dose tablet formulation (4.5 mg) of a selective, nonergot, and D2-like R agonist (pramipexole). The data reported in this paper describes our clinical observations in a clinical trial setting.

The trail was conducted in compliance with the study protocol, Declaration of Helsinki, and current GCP guidelines, as well as the other applicable national and international regulatory requirements and was approved by the independent Ethics Committee, of the “Evaluation of Ethics in Biomedical Research” (BEBO) Foundation, Assen, the Netherlands.

The medical history (including smoking, alcohol, and drug abuse), physical examinations, thorough laboratory investigations, and ECG assessments indicated the mental and physical healthy states of all participants. Written informed consent was obtained from all 52 subjects before initiation of any study-related procedure.

The test medication was administered orally over 30 days subdivided into 3 phases. The first phase consisted of thirteen uptitration days in which test drug was administered (under fasting conditions) in an escalating dose level design of 0.125 mg per day ascending up to and including 4.5 mg as previously described by Jansen and associates [10]. This was followed by a 12-day steady-state phase in which the test drug was orally administered as a single daily oral dose of 4.5 mg under fasting conditions. The last phase consisted of 5 downtitration days in which the dose was deescalated down to 0.75 mg a day. A poststudy visit was conducted 3–5 days after the last dose.

All study participants were given oral domperidone (10 mg TID) to prevent potential dopamine agonist-peripheral effects such as OH, nausea, and vomiting.

Brachial systolic and diastolic blood pressure (SBP and DBP), heart rate (HR), and ECG recordings were collected and assessed at 2 h after dose time points. Additional assessments (including manual assessment of the vital signs) for safety purposes were obtained when deemed necessary. Assessments were done noninvasively using Philips IntelliVue patient monitor; MP40/50, version B1. Brachial SBP, DBP, and HR were assessed in supine position after lying in bed for 5 minutes and after standing for 3 minutes.

For this specific publication, the following derived parameters were assessed as these are considered to be most meaningful to express cardiovascular influences: pulse pressure (PP) equal to SBP minus DBP; pulsatile stress (PS) equal to HR multiplied by PP; and resting rate pressure product (RPP) defined as SBP multiplied by HR.

## 3. Statistics

Data were available from 52 subjects. Data obtained from one subject have not been included in the statistical analysis of this report due to unreliability of collected vital signs results. This subject developed recurrent vasovagal signs and symptoms during blood sampling.

Fifty-one study participants were categorized into 3 age groups, G1 (24 subjects, aged 18–29 years), G2 (13 subjects, aged 30–40 years), and G3 (14 subjects, aged 40–55 years). Supine and standing BP, HR, and derived parameters data obtained from the 3 age groups during different phases of the clinical trial were analysed and compared using a repeated measures ANCOVA, with the baseline (BL) values as covariate. Analysis included values obtained at screening (BL) and during uptitration phase (UPT), steady-state phase (STS), downtitration phase (DNT), and follow-up (FU) visit of the clinical trial, in both supine and standing positions.

In addition, we investigated the presence of dose-dependent change (trend) in BP, HR, and derived parameters levels during the 13-day UPT phase. For this analysis a repeated measures analysis was used, and estimates were created for comparison of the results of UPT days 2 until 13 versus day 1, to assess from which time point differences with the first measurement were apparent. Furthermore, contrasts were defined to obtain an indication of a linear trend through time. No tests for higher-order trends have been performed. For none of the statistical analyses correction for multiple comparisons was incorporated, as the results are considered explorative and as such are meant to set the stage for further confirmative research. All statistical analyses were done using SAS version 9.1.3. All found *P* values are two-sided and alpha = 0.05 has been used.

## 4. Results

**Figure 1:**
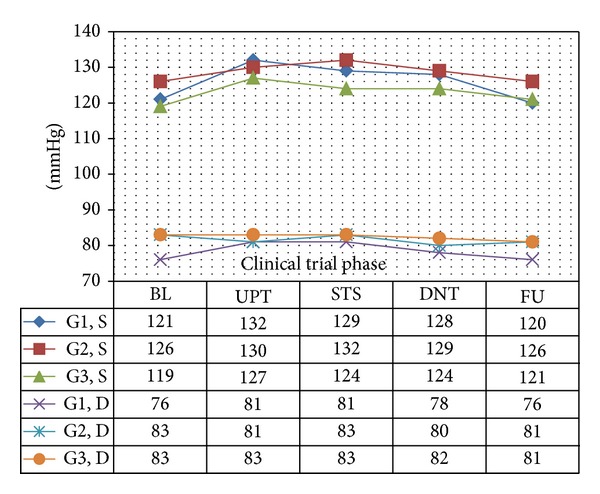
Means of resting supine systolic blood pressure (S) and diastolic blood pressure (D). Significance tests: (1) SBP-UPT versus BL, *P* < 0.0001; STS versus BL, *P* < 0.0001. (2) DBP-UPT versus BL, *P* = 0.0310; STS versus BL, *P* = 0.1171.

**Figure 2:**
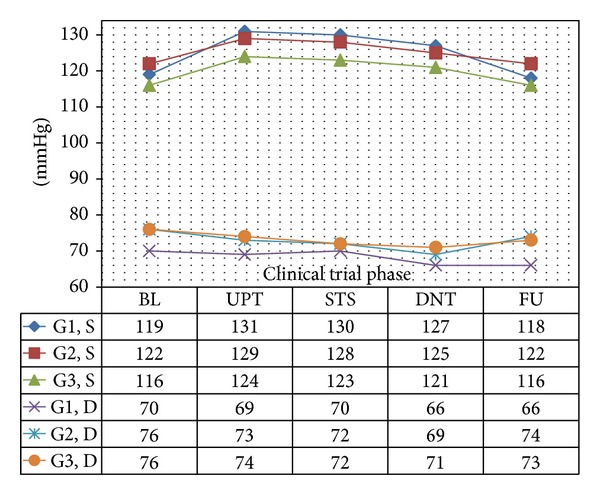
Means of resting standing systolic and diastolic blood pressure. S: systolic blood pressure, D: diastolic blood pressure; Significance tests: (1) SBP-UPT versus BL,  *P* < 0.0001; STS versus BL, *P* < 0.0001. (2) DBP-UPT versus BL, *P* = 0.09; STS versus BL, *P* = 0.02.

**Figure 3:**
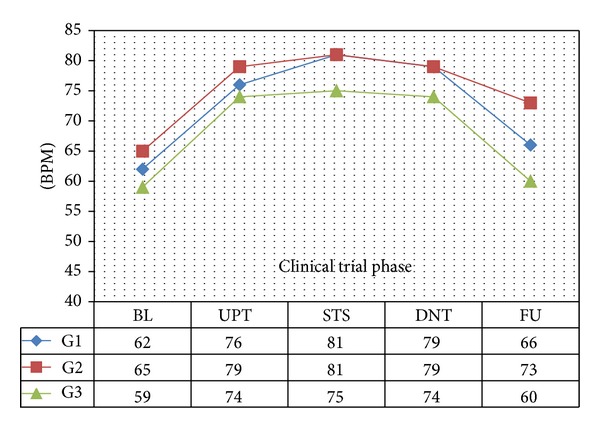
Mean resting supine heart rate. BPM: beat per minute; significance tests: UPT versus BL, *P* < 0.0001; STS versus BL, *P* < 0.0001.

**Figure 4:**
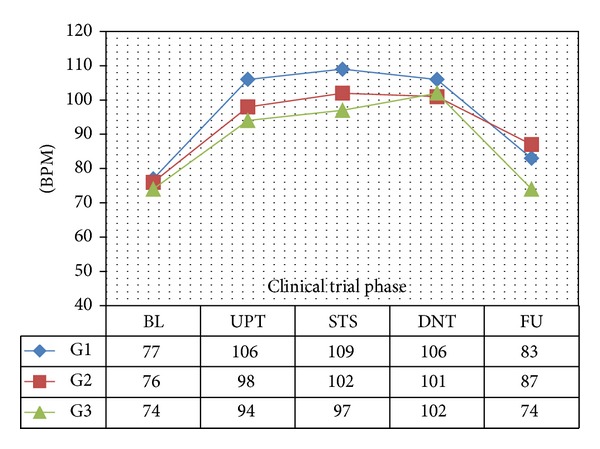
Mean resting standing heart rate. BPM: beat per minute; significance tests: UPT versus BL, *P* < 0.0001; STS versus BL, *P* < 0.0001.

**Figure 5:**
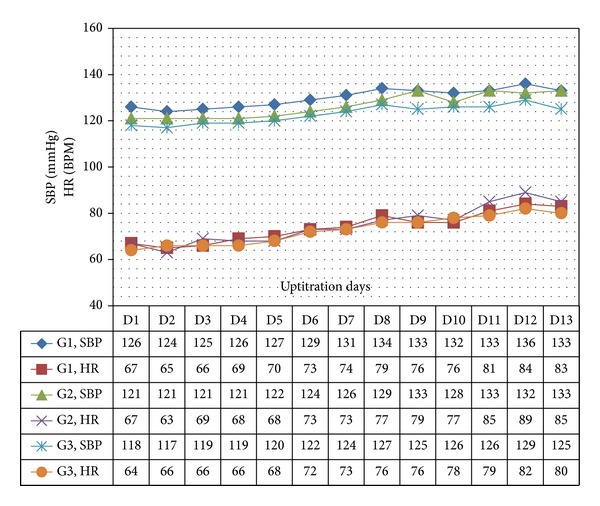
Trends in supine systolic blood pressure and heart rate over 13 uptitration days. SBP: systolic blood pressure, HR: heart rate, BPM: beat per minute.

**Figure 6:**
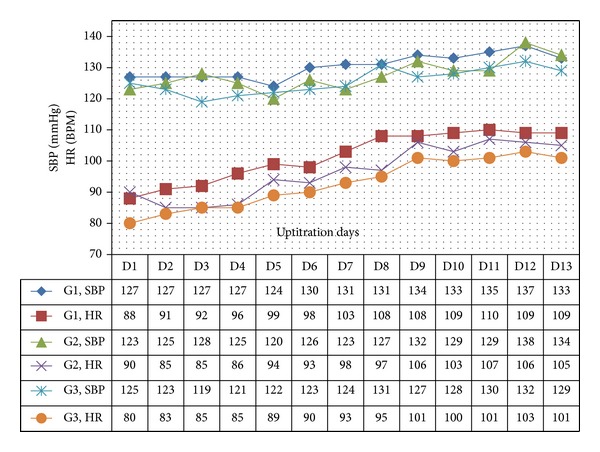
Trends in standing systolic blood presure and heart rate over 13 uptitration days. SBP: systolic blood pressure, HR: heart rate, BPM: beat per minute.

**Figure 7:**
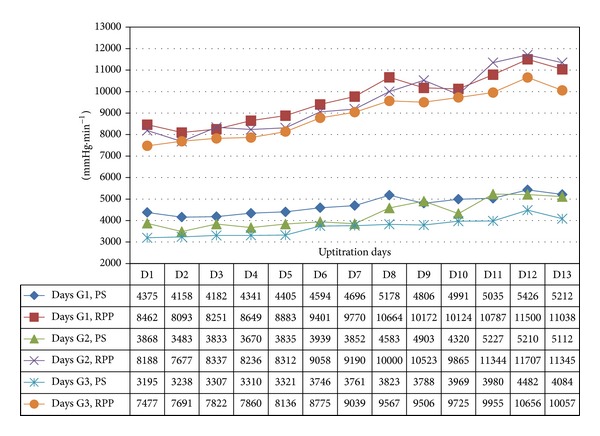
Trends in supine pulsatile stress (PS) and rate pressure product (RPP) over 13 uptitration days.

**Figure 8:**
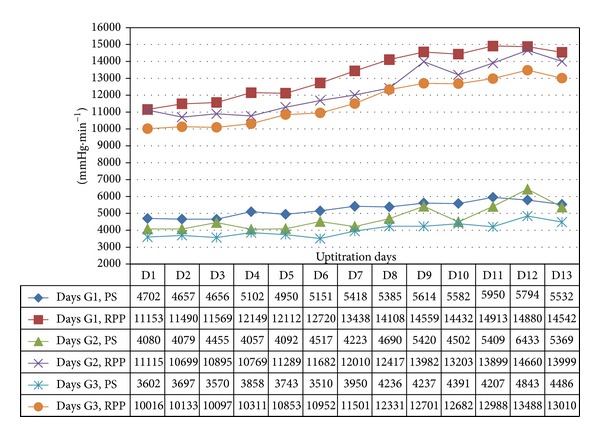
Trends in standing pulsatile stress (PS) and rate pressure product (RPP) over 13 uptitration days.

In all 3 age categories, we found clinically relevant and statistically significant increases in the mean values of SBP, HR, PP, PS, and RPP obtained during UPT and STS phases as compared to BL line values. The increases were more apparent for daily doses of 1.5 mg and higher. Regarding DBP, values obtained from G1 showed 5 mmHg increases in supine values obtained during both UPT and STS phases. Values obtained in standing position showed no relevant changes from BL. G2 and G3 demonstrated no relevant changes in values obtained in supine position, while both groups demonstrated 2–4 mmHg declines in standing values as compared to BL. Figures 1, 2, 3, and 4 and Tables 1, 2, 3, 4, 5, and 6 demonstrate the obtained results. In addition, we found dose-related increases in SBP, HR, and all derived parameters, except for PP values in G1 and G3, during the 13-day UPT phase. The statistical analysis results suggest that a linear trend exists: differences compared to BL gradually became larger, and starting from Day 5 to Day 9 depending on the parameter the corresponding *P* values became statistically significant. Figures 5, 6, 7, and 8 demonstrate the observed trends in supine and standing SBP, HR, PS, and RPP.

During the trial, 10 subjects (6 of G1, 25%, and 4 of G2, 31%) were removed prematurely from the trial because of clinically significant changes in the vital signs associated with neuromuscular symptoms. One of these subjects (belonging to G2) demonstrated orthostatic intolerance with postural decline of 43 mmHg and 22 mmHg, respectively, in SBP and DBP, approximately 3.5 h after having received a 4.5 mg dose. This OH was associated with dizziness, diaphoresis, and generalized body tingling sensation. A concurrently obtained ECG tracing showed nonspecific ST segment depression in lower extremity leads, II, III, and aVF. During the event, supine BP and HR values obtained from this subject were higher than those obtained at BL. Supine and standing BP and (HR) were 145/80 mmHg (82 bpm) and 102/58 mmHg (111 bpm), respectively, as compared to 127/66 mmHg (77 bpm) and 128/79 (95 bpm) at BL. The subject was then given an IV dose of metoclopramide (dopamine antagonist) which was followed by correction of the orthostatic intolerance. The other 9 subjects demonstrated rapid symptomatic increase in SBP and HR during the UPT phase, after having received a daily dose of 1.5 mg (2 subjects), 2 mg (5 subjects), 3 mg (1 subject), and 4.5 mg (1 subject). Increases up to 54 mmHg and 81 mmHg above baseline values have been encountered, respectively, in supine and standing SBP. In addition, increases up to 64 bpm and 179 bpm above BL, respectively, in supine and standing HR have been observed in these subjects. The changes in vital signs were associated with the following symptoms and signs: rapidly progressing sustained lower limbs tremors in all 9 subjects, abdominal wall tremors in 6 subjects, and generalized body tremors in 2 subjects. In 1 subject, the generalized body tremors were associated with sinus tachycardia of 239 bpm (compared to a normal regular heart rate of 60 bpm at BL), restlessness, reduced concentration, blurred vision, and slow, involuntary, intermittent, and uncontrollable lower limb movements. Other associated symptoms and signs included intolerable pulsating temporal and ocular headache in 3 subjects. In 1 of these 3 subjects, an intermittent uncontrollable lower limb movement has been observed. Three subjects reported dyspnoea, one subject reported feeling of chest compression, 1 subject reported an intermittent precordial pain, and in one subject bilateral dilated poorly reactive pupils have been observed. In 4 of the 9 subjects nonspecific St-segment depression was seen in the inferior limb leads of concurrently obtained ECG. The subjects clinical condition required immediate administration of oxygen and intravenous (IV) rescue medications to all subjects including a selective beta-1 (*β*-1) blocking agent, metoprolol, and dose ranging from 10 to 15 mg. This was followed by oral metoprolol at a dosage of 50 once daily for 1-2 days. One subject had an inadequate response to the IV selective *β*-1 blocking agent. This subject demonstrated rapid fluctuating increases in BP values, as high as 35 mmHg and 52 mmHg above obtained BL values, respectively, in supine and standing BP (resp., 173/72 mmHg and 201/90 mmHg versus BL values of 138/79 mmHg and 149/84 mmHg). This was associated with palpitation, chest compression, diaphoresis, and generalized body shakiness. In this subject a continuous IV infusion with an alpha and beta blocking agent, labetalol, in a dose of 87.5 mg was administered as add-on therapy. During medical treatment, BP and cardiac electrical activity (using cardiac telemetry) of all subjects were closely monitored. To rule out any high blood pressure-induced cardiac pathology, cardiac enzymes (cardiac troponin and CK-MB) have been assessed in subjects with St-segment changes. All obtained values were normal.

In another 5 patients, some episodes of OH have been observed. These episodes were of short duration, nonpersistent, and could be treated conservatively without pharmacological intervention and therefore did not necessitate the withdrawal of these 5 subjects from the study.

## 5. Discussion

Dopamine D2 receptor agonists are a first line treatment option in young Parkinson's patients with mild-to-moderate symptoms [7]. Beside their central nervous system effect, dopamine agonists modulate the functions of other body systems including CV system (S). Selective stimulation of D2 receptors induces bradycardia and lowers BP in human and animals [2, 11–13]. Earlier reports [11, 14] demonstrated OH in 30–56% of PD patients receiving D2R agonists, both ergot and nonergot derivatives. Moreover, the administration of escalating doses of D2R agonist, 0.5, 1.0, and 2.0 mg per day to patients with essential hypertension, induced dose proportional decreases in BP [15]. Peters et al. [16] studied the CVS effect of a selective nonergot D2R agonist in healthy male volunteers. The volunteers received escalating doses of 0.125 mg, 0.25 mg, 0.5 mg, 0.75 mg, and 1 mg TID, each for 3-day period. Drug-induced symptomatic OH with moderate increase in average HR has been found in 63% of subjects. No blood pressure elevating effect has been reported by the authors.

In this report, we demonstrated elevations in SBP, HR, PP, PS, and RPP, in both supine and standing positions, after the administration of a selective D2R agonist, pramipexole in healthy male subjects. Although vital signs changes were more pronounced in subjects younger than 30 years of age, changes observed in subjects aged 40 years or older might be more interesting. The reason of this is that although PD develops in approximately one third of patients before the age of 50 [17], the disease commences before the age of 40 in only 5% of patients [18]. This together with the fact that the usual D2R agonist doses in PD patients are above 1.5 mg per day underscores the clinical relevance of our findings.

The encountered drug-induced supraphysiologic increases in vital signs may explain the reported D2R agonist treatment associated CV morbidity in PD patients such as HF [9]. In a meta-analysis of individual data obtained from one million adults, long term increase of 10 mmHg in SBP was found to be associated with 30% higher risk of death from coronary heart disease (CHD). Even an increase of 2 mmHg in SBP can be associated with 7% mortality from ischemic heart disease [19, 20].

High resting (R) HR is an independent strong predictor of CV morbidity (including CHD and HF) and mortality in both healthy individuals [21–24] and hypertensive patients [25]. Population based studies indicated that every 20 bpm increase of the RHR is associated with 30–50% increase in CV mortality [24]. Benetos et al. [22] found a significant increase in CV mortality due to CHD among general French population who demonstrated resting supine HR between 61 and 80 bpm. The risk progressively increased with increasing HR > 80 bpm. In a more recent study, Cooney et al. [23] demonstrated a strong graded independent relationship between RHR and CV mortality in healthy male and female subjects. The hazard ratio for CV mortality was 1.24 and 1.32, respectively, in men and women for each 15 bpm increase in RHR. The association between increased HR and CV morbidity and mortality has been attributed to increase in myocardial oxygen demand and energy utilization together with reduced diastolic coronary perfusion time [21]. This leads to discordance between increased myocardial oxygen supply and demand resulting in cardiac ischaemic events.

Increase in PP has also been reported to be an independent predictor of CV morbidity and mortality due to coronary artery disease, myocardial infarction, and congestive HF in male and female subjects [26–29]. In normotensive subjects, each 10 mmHg elevation in PP above BL (as observed in values obtained in supine position, in this report) has been found to be associated with 14–21% increase in risk of CHF and CV mortality [27, 29]. The two major determinants of PP are cardiac stroke volume and compliance of arterial tree. In this report, the intrasubject increase in PP can be explained by the observed increase in SBP (reflecting an increase in stroke volume) without relevant change in DPB. A sustained supraphysiologic elevation in PP and HR as observed in subjects aged 40 years or over (approximately 50% increase in resting supine and standing HR × PP double product) leads to large vessel atherosclerosis, increased vessel stiffness, and reduced arterial compliance that will further amplify PP. The resultant clinical sequelae can be translated from the biological impact of heightened resting PS on the vascular wall. Under normal conditions arterial vascular walls are continuously exposed to physiologic levels of cyclic strains and pulsatile distension imposed by stroke volume and systolic-diastolic blood pressure phases. Normal physiologic variation in arterial wall pulsatile distension does not exceed 10–12% [19]. Non-physiologic increased chronic PS as indicated in this report (≥51% in supine position and ≥47% in standing position) stimulates the expression and activity of a number of vessel wall proteolytic enzymes that cleave extracellular matrix as well as nonmatrix substances resulting in increased permeability to macromolecules including LDL. Cyclic strain also increases reactive oxygen species generation and cleaved caspase expression, a proapoptotic event. These changes result in endothelial cell dysfunction, detachment, and apoptosis [19]. In addition, nonphysiologic increased cyclic stretch promotes vascular smooth muscle cell-DNA synthesis and proliferation leading to increased wall thickness and decreased vascular wall compliance [19]. This will further amplify PP and therefore initiates a vicious cycle, increased PP-reduced compliance [26]. The effect of this vicious circle is augmented by aging factor which is known to progressively reduce arterial compliance [30, 31].

We also assessed the resting RPP which is an index of cardiac load and myocardial oxygen consumption [30]. The observed increase in resting RPP, ≥32% above the obtained BL v

alues, mirrors increased sympathetic-induced myocardial metabolic demand with increase in coronary blood flow and myocardial oxygen consumption to meet the increased demand [32]. Given the fact that maximum cardiac reserve capacity decreases with age [33, 34], increased resting RPP may further limit the cardiac reserve capacity and therefore lower the threshold for HF in elderly subjects.

The changes in vital signs and derived parameters as well as the clinically significant elevation in SBP and or HR in about 18% of the 51 study participants could be attributed to stimulation of cardiac *β*-1 adrenergic receptors. Dopamine in moderate doses stimulates cardiac *β*-1 adrenoreceptors resulting in a positive inotropic effect and a vasodilation that manifest, itself clinically as an increase in SBP with insignificant change in DBP [35]. This line of reasoning explains our findings of test drug induced clinically relevant vital signs changes starting on days 6-7 of the UPT period onwards (1.5–2 mg/day). It may also call the attention to the value of concomitant use of selective *β*-1 blocking agents in clinical settings involving long-term treatment with dopamine agonists. This is particularly important in patients with PD where the effect of D2R agonists on cardiac chronotropicity and inotropicity may even become augmented. PD patients with OH (about 30–40% of PD patients) have cardiac and extracardiac sympathetic denervation, while those without OH can have normal innervation [36]. Cardiac sympathetic denervation in PD patients has been reported to be associated with supersensitivity of cardiac beta-adrenoreceptors [37, 38]. The use of directly acting beta-adrenoceptor agonist, isoproterenol, in patients with PD and OH led to exaggerated cardiac inotropic and chronotropic responses [36]. In another report [38] noradrenaline dose required for a 25 mmHg increase in SBP was significantly lower in patients with PD and OH as compared to control or PD patients without cardiac sympathetic denervation. The beneficial effect of lowering persistently elevated cardiac ino- and chronotropicity in reducing cardiovascular morbidity and mortality has been emphasized in the literature [19–21].

Finally, on all days of the clinical trial, domperidone was given orally in doses of 30 mg daily to prevent potential dopamine agonist-peripheral effects such as OH, nausea, and vomiting. A potential effect of domperidone on the observed vital signs changes in this study is very unlikely. Earlier reports [39, 40] indicated that pharmacological action of domperidone is mediated through peripheral dopamine receptors (D1 and D2 receptors) and *α*-adrenergic receptors (*α*-1 and *α*-2 adrenoreceptors) and that domperidone antagonizes noradrenaline- and dopamine-induced smooth muscle relaxations by selectively inhibiting *α*_1_-adrenoceptors [39]. In addition, Martinez-Mir et al. [41] studied the cardiac effect of domperidone in guinea-pig atria. Dopmeridone showed a negative inotropic effect and failed to modify cardiac chronotropic responses elicited by dopamine and noradrenaline. In this report, the increase in SBP was associated with no or even mild decline in DBP in G2 and 3 subjects. Alpha adrenergic blocking effect would be expected to elevate the peripheral resistance and results in increase in DBP. Also, in 8 of the 9 subjects with severe increase in SBP and or HR administration of a *β*-1 blocking agent led to normalization of BP and HR. This ruled out potential confounding effects of domperidone on the observed vital sign changes.

In conclusion, Dopamine D2 agonist is a preferred first line option in treatment of young PD patients. Treatment-associated clinically significant increases in HR, SBP, amplitude and frequency of PS, and resting RPP, as demonstrated in this report, bear clinical relevance and might explain some of the cardiovascular morbidity (e.g., HF) observed in patients receiving dopamine D2R agonist in clinical settings. The use of water soluble *β*-1 adrenergic blocking agents that poorly cross the blood brain barrier and exhibit no intrinsic sympathomimetic activity might reduce the CV morbidity and mortality without exerting CNS side effects among PD patients on long-term D2R agonist therapy. Further clinical studies with PD patients are warranted to assess the effects of cardioselective *β*-1-blocker as add on therapy in patients with PD.

## Figures and Tables

**Table 1:** Supine pulse pressure in mm Hg (mean ± SD) in the 3 age groups.

Phase	G1	%^#^	G2	%^#^	G3	Versus BL
BL	50 ± 8		46 ± 7		40 ± 6	
UPT*	62 ± 10	24%	56 ± 9	22%	50 ± 10	25%
STS*	60 ± 10	20%	56 ± 7	22%	50 ± 11	25%
DNT	61 ± 11	22%	56 ± 6	22%	50 ± 11	25%
FU	52 ± 8	4%	48 ± 6	4%	43 ± 8	8%

*P < 0.0001 versus BL; ^#^percentage increase as compared to baseline.

**Table 2:** Standing pulse pressure in mm Hg (mean ± SD) in the 3 age groups.

Phase	G1	%^#^	G2	%^#^	G3	Versus BL
BL	45 ± 9		43 ± 6		37 ± 8	
UPT*	51 ± 12	13%	49 ± 10	14%	44 ± 11	19%
STS*	49 ± 11	9%	49 ± 10	14%	41 ± 12	11%
DNT	50 ± 11	11%	50 ± 8	16%	42 ± 12	14%
FU	44 ± 8	−2%	45 ± 10	5%	40 ± 11	8%

*P < 0.0007, versus BL; ^#^percentage increase as compared to baseline.

**Table 3:** Supine pulsatile stress in mm Hg·Min^−1^ (mean ± SD) in the 3 age groups.

Phase	G1	%^#^	G2	%^#^	G3	Versus BL
BL	3068 ± 729		2999 ± 699		2360 ± 310	
UPT*	4471 ± 1190	46%	4411 ± 1258	47%	3677 ± 874	56%
STS*	4893 ± 1094	59%	4531 ± 858	51%	3752 ± 802	59%
DNT*	4753 ± 871	55%	4442 ± 799	48%	3976 ± 942	68%
FU	3477 ± 934	13%	3491 ± 846	16%	2598 ± 571	10%

*P < 0.0001, versus BL; ^#^percentage increase as compared to baseline.

**Table 4:** Standing pulsatile stress in mm Hg·Min^−1^ (mean ± SD) in the 3 age groups.

Phase	G1	%^#^	G2	%^#^	G3	Versus BL
BL	3464 ± 864		3268 ± 754		2685 ± 563	
UPT*	5338 ± 1508	54%	4861 ± 1384	49%	4047 ± 1049	51%
STS*	5294 ± 1301	53%	4953 ± 1115	52%	3935 ± 1067	47%
DNT*	5246 ± 1176	51%	5043 ± 1076	54%	4215 ± 1138	57%
FU	3676 ± 909	6%	3997 ± 1404	22%	2866 ± 474	7%

*P < 0.0001, versus BL; ^#^percentage increase as compared to baseline.

**Table 5:** Supine resting rate pressure product in mm Hg·min^−1^ (mean ± SD) in the 3 age groups.

Phase	G1	%^#^	G2	%^#^	G3	Versus BL
BL	7385 ± 1542		7931 ± 1680		6915 ± 892	
UPT*	10070 ± 2225	36%	10156 ± 2844	28%	9143 ± 1881	32%
STS*	10573 ± 1845	43%	10393 ± 1650	31%	9150 ± 1341	32%
DNT*	9941 ± 1419	35%	9895 ± 1690	24%	9620 ± 1702	39%
FU	7855 ± 1569	6%	8903 ± 2162	12%	7025 ± 989	2%

*P < 0.0001, versus BL; ^#^percentage increase as compared to baseline.

**Table 6:** Standing resting rate pressure product in mm Hg·Min^−1^ (mean ± SD) in the 3 age groups.

Phase	G1	%^#^	G2	%^#^	G3	%
BL	9333 ± 1817		9575 ± 1990		8743 ± 1134	
UPT*	13878 ± 2789	49	12866 ± 3010	34%	11836 ± 2070	35
STS*	14080 ± 1833	51	13421 ± 1586	40%	12003 ± 1633	37
DNT*	13503 ± 1852	45	13111 ± 2201	37%	12570 ± 1649	44
FU	10012 ± 1772	7	11092 ± 2920	16%	8790 ± 810	0.05

*P < 0.0001, versus BL; ^#^percentage increase as compared to baseline.
